# The Role of the Eukaryotic Translation Initiation Factor 4E (eIF4E) in Neuropsychiatric Disorders

**DOI:** 10.3389/fgene.2018.00561

**Published:** 2018-11-23

**Authors:** Inês S. Amorim, Gilliard Lach, Christos G. Gkogkas

**Affiliations:** ^1^Centre for Discovery Brain Sciences, The University of Edinburgh, Edinburgh, United Kingdom; ^2^The Patrick Wild Centre, The University of Edinburgh, Edinburgh, United Kingdom

**Keywords:** eIF4E, neurodevelopmental/neuropsychiatric disorders, anxiety, depression, protein synthesis, translation, Autism Spectrum Disorders, Fragile X Syndrome

## Abstract

Protein synthesis in eukaryotic cells is a complex, multi-step and tightly regulated process. Translation initiation, the rate limiting step in protein synthesis, is dependent on the activity of eukaryotic translation Initiation Factor 4E (eIF4E). eIF4E is the cap-binding protein which, in synergy with proteins such as the helicase eIF4A and the scaffolding protein eIF4G, binds to mRNA, allowing the recruitment of ribosomes and translation initiation. The function of eIF4E is tightly regulated in cells under normal physiological conditions and can be controlled by post-translational modifications, such as phosphorylation, and by the binding of inhibitory proteins, including eIF4E binding proteins (4E-BPs) and CYFIP1. Recent studies have highlighted the importance of eIF4E in normal or aberrant function of the nervous system. In this mini-review, we will highlight the role of eIF4E function and regulation in the pathophysiology of neurodevelopmental and neuropsychiatric disorders.

## Introduction

**FIGURE 1:**
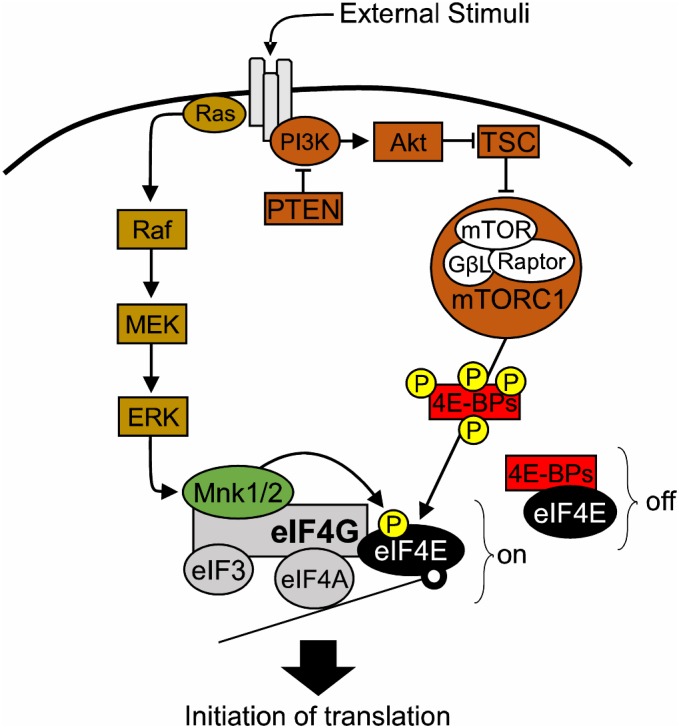
The role of eIF4E in translational control. Diagram of the major signaling pathways upstream of eIF4E. 4E-BPs, eIF4E-binding proteins; Akt, also known as Protein kinase B (PKB); eIF3, eukaryotic initiation factor 3; eIF4A, eukaryotic translation initiation factor 4A; eIF4E, eukaryotic translation initiation factor 4E; eIF4G, eukaryotic translation initiation factor 4G; ERK, extracellular signal–regulated kinase, also known as mitogen-activated protein kinase (MAPK); GβL, G protein beta subunit-like; MEK: mitogen-activated protein kinase kinase; MNK1/2, mitogen-activated protein (MAP) kinase-interacting serine/threonine-protein kinases 1/2; mTOR, mechanistic target of rapamycin; mTORC1, mechanistic target of rapamycin complex 1; off, repression of translation; on, active translation; P, phosphorylation site; PI3K, phosphoinositide 3-kinase; PTEN, phosphatase and tensin homolog; RAPTOR, regulatory-associated protein of mTOR; TSC1/2, Tuberous sclerosis proteins 1/2.

In Archaea and Bacteria, the mRNA Shine-Dalgarno sequence promotes binding of the ribosome to mRNA and thus translation initiates (Shine and Dalgarno, 1975). Contrary to that, the majority of eukaryotic precursor mRNAs harbor a 5′ end cap, a 7 methylguanosine triphosphate (m7GpppG) structure, which serves as a docking point for eukaryotic translation initiation factors (eIFs) (Furuichi and Miura, 1975; Shatkin, 1976; Furuichi et al., 1977; Sonenberg et al., 1979). eIF4E directly binds the mRNA 5′ cap (Sonenberg et al., 1979) and interacts with the scaffolding protein eIF4G, which in turn binds the helicase eIF4A to form the eIF4F complex and allow the recruitment of ribosomes to initiate the predominant form of eukaryotic translation: cap-dependent translation (Gingras et al., 1999). eIF4G provides the backbone of the eIF4F complex. In addition, it helps to circularize mRNAs through its interaction with poly(A) binding proteins (PABPs) and provides a binding site for other regulatory factors, such as MAP kinase-interacting serine/threonine-protein kinases 1/2 (MNK1/2) and eukaryotic Initiation Factor 3 (eIF3). The eIF4A helicase unwinds secondary structures present in the mRNA 5′ Untranslated Regions (UTRs) to facilitate translation. eIF4A helicase activity is promoted by eIF4G and eIF4B (Gingras et al., 1999), as well as eIF4E (Feoktistova et al., 2013). Cap-dependent translation is responsible for the bulk of protein synthesis in eukaryotic cells, and is the rate limiting step in protein synthesis. Crucially, eIF4E and the eIF4F complex carry a well-conserved function in eukaryotes, where they preferentially regulate the synthesis of a subset of proteins, by controlling mRNA translation initiation (Pelletier and Sonenberg, 1987; Sonenberg and Hinnebusch, 2007, 2009; Hinnebusch et al., 2016). Thus, the term “eIF4E/eIF4F-sensitive” mRNAs has emerged (Hinnebusch et al., 2016). Consequently, eIF4E activity is tightly regulated by a variety of factors such as hormones, growth factors, cytokines and other extracellular stimuli, which converge on two major signaling cascades: MAPK/ERK and PI3K/mTOR pathways (Joshi and Platanias, 2014; Saxton and Sabatini, 2017; Figure 1).

The PI3K/mTOR pathway regulates eIF4E function via the action of eIF4E-binding proteins (4E-BPs) (Pause et al., 1994). 4E-BPs compete with eIF4G for binding on the dorsal surface of eIF4E, hence disrupting the formation of the eIF4F complex (Pause et al., 1994). Hypo-phosphorylated 4E-BPs have a higher affinity for eIF4E and thus repress translation initiation (Gingras et al., 2001). Conversely, activation of PI3K/mTOR signaling leading to downstream phosphorylation of 4E-BPs by mTORC1, triggers the release of 4E-BPs from eIF4E. As a result, the availability of eIF4E for initiation increases (Haghighat et al., 1995; Richter and Sonenberg, 2005).

Activation of MAPK/ERK pathway leads to phosphorylation of eIF4E at the Serine 209 residue by MNK1/2 (Joshi et al., 1995; Ueda et al., 2004). MNK1/2 are recruited to the eIF4F complex by binding to the c-terminal domain of eIF4G, where they promote the phosphorylation of eIF4E (Pyronnet et al., 1999; Shveygert et al., 2010). While two studies suggested that eIF4E phosphorylation is either not required for translation (McKendrick et al., 2001) or that it decreases cap-dependent translation (Knauf et al., 2001), the majority of the literature suggests that eIF4E phosphorylation promotes initiation (Pyronnet et al., 1999; Lachance et al., 2002; Panja et al., 2014; Bramham et al., 2016). Moreover, several studies have identified phospho-eIF4E-sensitive mRNAs in cancer (Furic et al., 2010; Grzmil et al., 2011; Konicek et al., 2011; Robichaud et al., 2015) and in the nervous system (Gkogkas et al., 2014; Cao et al., 2015; Amorim et al., 2018). eIF4F complex formation is also affected by sequestering eIF4E in a repressive complex with the Fragile X Mental Retardation Protein (FMRP) and the Cytoplasmic FMRP Interacting Protein (CYFIP1) (Napoli et al., 2008). The eIF4E-CYFIP complex is sensitive to MNK1/2 activity and precludes eIF4E-eIF4G binding, thus hindering translation initiation (Napoli et al., 2008; Panja et al., 2014; Genheden et al., 2015; Bramham et al., 2016).

**FIGURE 2:**
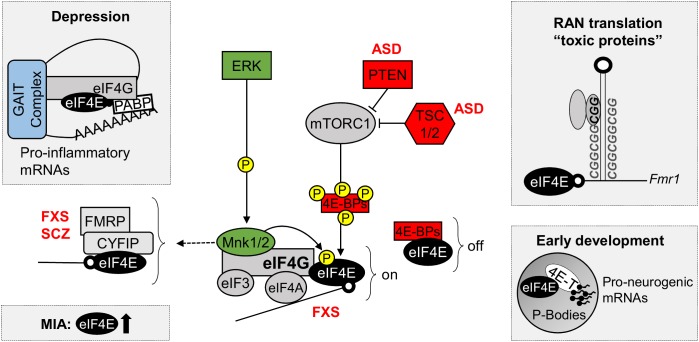
eIF4E in neurodevelopmental and neuropsychiatric disorders. Diagram of the control of eIF4E function by ERK and mTOR signaling pathways, highlighting the new roles of eIF4E in depression, maternal immune activation (MIA), repeat-associated non-AUG (RAN) translation and in early development. Top left: Decreased phosphorylation of eIF4E promotes depressive and anxiety behaviors by relieving translational repression of pro-inflammatory mRNAs containing repressive 3′ GAIT elements of translation; Bottom left: eIF4E and mTOR are dysregulated in gene expression profiles following MIA, a risk factor for the development ASD; Top right: RAN translation, a process important for the synthesis of toxic polypeptides containing poly nucleotide repeats, such as FMRP, requires functional eIF4E; Bottom right: eIF4E interacts with 4E-T in P-bodies, where they sequester and repress the translation of pro-neurogenic mRNAs during early development. 4E-BPs, eIF4E-binding proteins; 4E-T, eIF4E transporter; ASD, Autism Spectrum Disorders; CYFIP1, cytoplasmic FMRP interacting protein; eIF3, eukaryotic initiation factor 3; eIF4A, eukaryotic translation initiation factor 4A; eIF4E, eukaryotic translation initiation factor 4E; eIF4G, eukaryotic translation initiation factor 4G; ERK, extracellular signal–regulated kinase, also known as mitogen-activated protein kinase (MAPK); FMRP, Fragile X mental retardation protein; FXS, Fragile X Syndrome; GAIT complex, interferon (IFN)-γ-activated inhibitor of translation complex; MNK 1/2, mitogen-activated protein (MAP) kinase-interacting serine/threonine-protein kinases 1/2; MIA, maternal immune activation; mTORC1, mechanistic target of rapamycin complex 1; off, repression of translation; on, active translation; P, phosphorylation site; PABPs, poly-A binding proteins; PTEN, phosphatase and tensin homolog; RAN, repeat-associated non-AUG; SCZ, Schizophrenia; TSC1/2, Tuberous sclerosis proteins 1/2.

eIF4E-dependent translational control is linked to several cellular processes, including cell cycle progression, cell survival, cell motility and tumorigenesis (Rhoads et al., 1993; Malka-Mahieu et al., 2017; Steinberger et al., 2017), as well as inflammation, immunity and viral infection (Jan et al., 2016; Hoang et al., 2018). Nevertheless, a growing body of evidence indicates eIF4E-dependent translation is important for neuronal cell function and implicates its aberrant function in nervous system disorders (Figure 2), such as neurodevelopmental and neuropsychiatric conditions (Costa-Mattioli and Monteggia, 2013; Santini and Klann, 2014; St Clair and Johnstone, 2018).

## Autism Spectrum Disorders (ASD) and Fragile X Syndrome (FXS)

Autism Spectrum Disorders are a group of heterogeneous neurodevelopmental conditions characterized by persistent deficits in sociability, impaired communication, and the presence of restricted or repetitive stereotypical behaviors (Elsabbagh et al., 2012). ASDs are amongst the most common neurodevelopmental disorders, with an estimated prevalence of 16:1000 in children aged 8 years in the United States and high heritability (Sandin et al., 2017; Baio et al., 2018). ASDs have been linked to a variety of genetic mutations and risk factors, including genetic variants in conditions such as Rett syndrome, Tuberous Sclerosis and FXS (Carter and Scherer, 2013). FXS is one of the most common inherited forms of intellectual disability and is the leading genetic cause of ASDs (Gabis et al., 2011; McCary and Roberts, 2013). FXS is caused by a mutation (CGG nucleotide repeats) in the 5′ UTR of the *FMR1* gene, leading to transcriptional silencing of *FMR1* and subsequent loss of FMRP expression (Santoro et al., 2012). FMRP is a translational repressor, thus its loss engenders an across the board increase in protein synthesis (Li et al., 2001). Protein synthesis-linked hyperactivation of type I metabotropic glutamate receptors (mGluRs), leading to synaptic dysfunction, constitutes the predominant mechanistic theory aiming to explain the diverse pathophysiology of FXS (Bear, 2005).

Likewise, ASD is believed to arise from common downstream defects in synaptic function and brain connectivity (Abrahams and Geschwind, 2008). A leading hypothesis posits that downstream defects in mRNA translation lead to aberrant local protein synthesis, which results in altered synaptic development and plasticity (Kelleher and Bear, 2008; Gkogkas and Sonenberg, 2013). In accordance, patients and animal models of ASD and FXS show widespread alterations in synaptic plasticity and dysregulated mRNA translation (Kelleher and Bear, 2008; Jung et al., 2014; Contractor et al., 2015; Dahlhaus, 2018). Altered translation in ASD and FXS results not only from mutations in genes that directly impact on translational control mechanisms, but also from altered signaling, upstream of translation (MAPK/ERK and PI3K/mTOR) (Costa-Mattioli and Monteggia, 2013). A pivotal convergence point of these pathways is the control of cap-dependent translation, particularly through the function of the eIF4F complex.

Abnormalities in the *EIF4E* locus have been identified in genetic studies of autistic patients (Yonan et al., 2003; Neves-Pereira et al., 2009; Waltes et al., 2014). Moreover, a comparison of gene-expression in rodent models of maternal immune activation (MIA) with ASD patient cortical gene-expression revealed a strong involvement of the Tsc2/mTOR/eIF4E axis (Lombardo et al., 2018). MIA during the first trimester of pregnancy increases the risk for ASD most likely by affecting fetal brain development. Notwithstanding some epidemiological evidence, there is no compelling, direct link of *EIF4E* to ASDs. Nonetheless, several reports from animal models of ASD provide strong evidence for a key role of *Eif4e* in ASD. Deletion of *Eif4ebp2* (the predominant 4E-BP in the brain) or overexpression of *Eif4e* in mice lead to altered synaptic excitation/inhibition balance and altered behaviors, such as social interaction deficits, altered ultrasonic vocalizations, and repetitive/stereotyped behaviors (Gkogkas et al., 2013; Santini et al., 2013). Molecular, electrophysiological and behavioral defects in mice, which are reminiscent of ASD phenotypes diagnosed in patients, could be normalized by inhibition of cap-dependent translation using 4EGI-1 (Gkogkas et al., 2013; Santini et al., 2013), a small molecule developed as an eIF4E-eIF4G interaction inhibitor (Moerke et al., 2007). Recent work revealed that type I mGluR agonists, which were proposed as FXS therapeutics, also rescue phenotypes reminiscent of ASD in *Eif4ebp2* knockout mice (Aguilar-Valles et al., 2015). Furthermore, 4EGI-1 has shown beneficial effects in *Fmr1*^-/y^ mice, a model of FXS, where it reversed contextual memory deficits and normalized altered dendritic morphology, dysregulated actin dynamics and exaggerated mGluR-dependent LTD (Santini et al., 2017). Interestingly, crossing *Fmr1*^-/y^ mice with *Eif4e* overexpressing mice engenders cognitive impairments in addition to ASD-like phenotypes (Huynh et al., 2015).

Regulation of eIF4E by phosphorylation is associated with FXS. Patients and animal models of FXS show increased levels of phosphorylated eIF4E (Hoeffer et al., 2012; Gkogkas et al., 2014; Sidhu et al., 2014). Moreover, genetic deletion of the MNK1/2 kinases, which phosphorylate eIF4E, administration of the MNK1/2 inhibitor cercosporamide, or substitution of the eIF4E phosphorylation site for a non-phosphorylatable residue (*Eif4e*^ki/ki^ mice; Ser209Ala), ameliorated FXS phenotypes in *Fmr1*^-/y^ mice (Gkogkas et al., 2014).

In addition to eIF4E phosphorylation, a new translational control mechanism was found where FMRP interacts with eIF4E through CYFIP1 to prevent eIF4E-eIF4G binding, thereby hindering translation initiation (Napoli et al., 2008). The FMRP-CYFIP1-eIF4E complex is present in dendritic spines and actively participates in the local control of protein synthesis during synaptic activity. Synaptic activation by BDNF or mGluR stimulates the release of FMRP and CYFIP1 from eIF4E and promotes local translation (Napoli et al., 2008; Genheden et al., 2015). In fact, the levels of CYFIP1 have been shown to influence the maturation of dendritic spines (Pathania et al., 2014; Oguro-Ando et al., 2015), whereas *Cyfip1*^+/-^ mice show some phenotypes similar to *Fmr1*^-/y^ mice, such as exaggerated mGluR-LTD (Bozdagi et al., 2012).

One avenue through which aberrant eIF4E-dependent translation may lead to the manifestation of ASDs is through the translational control of specific subsets of mRNAs. eIF4E was shown to regulate the translation of mRNAs with 5′ UTRs that are highly structured (Feoktistova et al., 2013; Hinnebusch et al., 2016) or that contain CERT (cytosine-enriched regulator of translation) motifs (Truitt et al., 2015). Furthermore, phosphorylation of eIF4E has been proposed to affect the translation of mRNAs containing 3′ GAIT (interferon (IFN)-γ-activated inhibitor of translation) elements (Amorim et al., 2018). Subsets of mRNAs controlled by eIF4E perform a variety of functions, such as promoting tumorigenesis or participating in the control of the circadian rhythm and serotonin pathways (Furic et al., 2010; Cao et al., 2015; Amorim et al., 2018). In addition, eIF4E-sensitive mRNAs encode scaffolding proteins such as neuroligins (Gkogkas et al., 2013; Pettem et al., 2013; Rothwell et al., 2014) and extracellular matrix components (Gkogkas et al., 2014; Amorim et al., 2018). Mutations in several neuroligin isoforms are present in ASD patients (Jamain et al., 2003). In addition, overproduction of neuroligins was shown to modulate synaptic function and behavior in animal models of ASD and FXS (Hines et al., 2008; Dahlhaus and El-Husseini, 2010; Dahlhaus et al., 2010; Gkogkas et al., 2013). Extracellular matrix metalloproteinase 9 (MMP-9) regulates spine morphology, synaptic plasticity, learning and memory (Huntley, 2012), and is implicated in phenotypes in rodent models of ASD and FXS (Bilousova et al., 2009; Rotschafer et al., 2012; Dziembowska et al., 2013; Sidhu et al., 2014). Translation of MMP-9 is stimulated by eIF4E phosphorylation, the levels of which are increased in FXS patients and *Fmr1*^-/y^ mice (Hoeffer et al., 2012; Gkogkas et al., 2014; Sidhu et al., 2014). In addition, modulation of MMP-9 expression in rodents modulates FXS phenotypes associated with increased eIF4E phosphorylation (Gkogkas et al., 2014; Gantois et al., 2017). Finally, a recent study proposes a novel mechanism, whereby eIF4E is required for repeat-associated non-AUG (RAN) translation of the *FMR1* gene (Kearse et al., 2016). CGG repeats in the *FMR1* gene stimulate RAN translation, which leads to the synthesis of toxic polypeptides (Todd et al., 2013).

Apart from direct translational control, eIF4E may be linked to ASD pathophysiology via a role in early neuronal development through its interaction with the eIF4E-Transporter (4E-T) in processing bodies (P-bodies), which are cytoplasmic granules involved in mRNA degradation (Eulalio et al., 2007). Here, eIF4E and 4E-T cooperate to sequester and repress the translation of pro-neurogenic mRNAs, such as transcription factors and neuronal differentiation-related mRNAs (Yang et al., 2014). In addition, recent work revealed that 4E-T also binds to Pumilio2 and that this complex ensures neuronal specification of deep and superficial layer murine cortical neurons (Zahr et al., 2018).

## Depression and Anxiety Disorders

Depressive and anxiety disorders are often comorbid and represent the most common causes of disability worldwide (Zhou et al., 2017). In addition, these psychiatric conditions are commonly present in people suffering from ASDs (Magnuson and Constantino, 2011). mTOR signaling is affected in patients with major depressive disorder as well as animal models of depression and anxiety (Jernigan et al., 2011; Chandran et al., 2013; Ignacio et al., 2016). Furthermore, treatment with selective serotonin reuptake inhibitors or other anti-depressant drugs, such as ketamine, were shown to affect mTOR and its downstream targets p70S6K and 4E-BP1 (Jernigan et al., 2011; Park et al., 2014; Liu et al., 2015; Zhuang et al., 2016; Abelaira et al., 2017). MAPK/ERK signaling is also altered in patients and animal models of depressive disorders (Dwivedi et al., 2001; Rao et al., 2007; Dwivedi and Zhang, 2016). Given the convergence of these two pathways in the control of translation initiation, the question arises of how eIF4E function may affect or be affected by depression and anxiety disorders.

Two recent studies revealed that mice with defective eIF4E phosphorylation (*Mnk1/2*^-/-^ or *Eif4e*^ki/ki^ mice) show behaviors reminiscent of depression and anxiety, concomitant with increased inflammatory responses. *Mnk1/2*^-/-^ and *Eif4e*^ki/ki^ mice displayed increased immobility in the force-swimming and tail suspension tests, increased latency to feed in a novelty suppressed feeding assay, and anxiety behaviors in the open-field and elevated plus maze tests (Aguilar-Valles et al., 2018; Amorim et al., 2018). Translational profiling in brain tissue from *Eif4e*^ki/ki^ mice revealed increased translation of genes involved in serotonin pathways concomitantly with reduced levels of serotonin in the brain (Amorim et al., 2018). Aguilar-Valles et al. (2018) further report impaired serotonin transmission in the prefrontal cortex and reduced firing of serotonergic neurons in the dorsal raphe of *Eif4e*^ki/ki^ and *Mnk1/2*^-/-^ mice. Moreover, loss of eIF4E phosphorylation resulted in elevated levels of key inflammatory cytokines, including TNFα, IFNγ, and IL-2, in addition to an exaggerated response to lipopolysaccharide-induced microglial activation and cytokine production (Aguilar-Valles et al., 2018; Amorim et al., 2018). Interestingly, administration of a dominant negative form of TNFα rescued the behavioral and electrophysiological abnormalities in *Eif4e*^ki/ki^ mice (Aguilar-Valles et al., 2018).

The connection between depression and inflammation has received increased attention (Miller and Raison, 2016). There is an elevated comorbidity between depression and chronic inflammatory conditions (Abbott et al., 2015; Euesden et al., 2017), and while several studies have found increased levels of pro-inflammatory cytokines in patients with anxiety and major depressive disorder (Dowlati et al., 2010; Shelton et al., 2011; Mostafavi et al., 2014; Michopoulos et al., 2015), anti-inflammatory drugs are effective in the treatment of depression (Müller, 2018; Wiedlocha et al., 2018). The new evidence from Amorim et al. (2018) suggests an important mechanism through which impaired translation control via dysregulated eIF4E phosphorylation downstream of the MAPK/ERK pathway affects the translation of specific mRNAs to directly influence the inflammatory response and impact on depression and anxiety-like behaviors. Amorim et al. (2018) propose that eIF4E phosphorylation may control inflammatory responses in depression by regulating binding of the 3′ UTR element (GAIT) onto the 5′ eIF4F complex in circularized pro-inflammatory mRNAs. The GAIT complex acts as a translational repression mechanism that controls the translation of pro-inflammatory mRNAs (Mukhopadhyay et al., 2009). Loss of eIF4E phosphorylation may decrease the affinity of the GAIT complex to eIF4F, thus allowing excessive translation of pro-inflammatory mRNAs.

## Schizophrenia (SCZ)

SCZ is a neuropsychiatric disorder characterized by a combination of positive, negative and cognitive symptoms, including hallucinations and delusions, apathy and social withdrawal, and attention, memory and executive thinking deficits, respectively. SCZ is a highly disabling condition, affecting around 1% of the population, and it has high heritability rates (Kahn et al., 2015). The etiology of SCZ is not fully understood and is associated with a variety of genetic and environmental risk factors. Even though *DISC1* is one of the better characterized genes regarding its association with SCZ (Roberts, 2007; Bradshaw and Porteous, 2012), several studies have additionally identified *de novo* copy number variants (CNVs) as conferring high risk for the development of the disease (Xu et al., 2008; Malhotra et al., 2011). Interestingly, these CNVs are often associated risk factors for other neurodevelopmental disorders, such as ASD, mental retardation and epilepsy (Sullivan et al., 2012). In addition, network analysis has suggested they affect neurodevelopmental, synaptic function and post-synaptic signaling pathways (Walsh et al., 2008; Kirov et al., 2012; Brennand et al., 2015).

One recurrent CNV associated with SCZ, as well as with cognitive and behavioral abnormalities, is the 15q11.2 microdeletion (Stefansson et al., 2008; Kirov et al., 2009; De Wolf et al., 2013). The 15q11.2 locus includes four genes – *CYFIP1*, *TUBGCP5*, *NIPA1*, and *NIPA2*. Of particular interest is the *CYFIP1* gene, which has emerged as an important player in ASD and FXS and participates in the control of protein synthesis and dendritic spine maturation. Furthermore, CYFIP1 is also part of the WAVE regulatory complex, which is involved in the control of actin polymerization and lamellipodia formation (Chen et al., 2010; Abekhoukh et al., 2017). The functioning of this complex and of CYFIP1 contribute to the correct formation of adherens junctions and cell polarity in patient-derived induced pluripotent stem cells (iPSCs) neuroprogenitor cells (Yoon et al., 2014). Other studies have used iPSCs-derived neuronal populations to address SCZ-related impairments in cell migration, cytoskeletal remodeling and protein synthesis (Brennand et al., 2015; Topol et al., 2015). Interestingly, by using olfactory neurosphere-derived cells, a model that was shown to replicate some of the molecular phenotypes of SCZ (Mackay-Sim, 2012), English et al. (2015) identified, both at the level of the transcriptome and proteome, significant changes in signaling pathways key to the control of mRNA translation, including eIF2, eIF4 and mTOR signaling. Although the role of mTOR in SCZ has not been well established, various studies have noted the presence of altered mTOR signaling, particularly in the context of *DISC1* animal models (Kim et al., 2009; Zhou et al., 2013; Gururajan and van den Buuse, 2014). Exploring the relationship between mTOR and SCZ will be particularly interesting in terms of its connection to eIF4E, given the ability of this initiation factor to interact with CYFIP1 and to influence crucial mechanisms to SCZ, such as dendritic spine morphology and neurodevelopment.

## Conclusion – Outlook

The genomic and gene-expression “boom” of the early 00’s, bolstered by the advent of technologies to measure transcriptional changes (micro-array, RNA/exome/whole genome next generation sequencing) has placed a focus on transcription as the key step in the gene-expression pathway underlying the pathophysiology of neuropsychiatric disorders (Kelsoe, 2004; Kavanagh et al., 2013; Nievergelt et al., 2018). Thus, the regulatory mechanisms of protein synthesis have not received much attention, while translational control and investigation of the translatome has not become part of large consortia mainly due to the lack of accessible genome-wide methodologies. The onset of translatome profiling will change this trend and important mechanistic data will surface in the coming years (Ingolia et al., 2009, 2018). Thirty-nine years after its discovery, eIF4E still poses an enigma as to the identity of the mRNAs it controls and the precise regulatory mechanisms it participates in during different stages of development and in different cell-types of the brain. Understanding the role of cap-dependent translation in the brain will facilitate the adaptation of the already existing compendium of biochemical/genetic models and pharmacological approaches to modulate translation (Sonenberg and Hinnebusch, 2007; Malina et al., 2011, 2012), for the treatment of neuropsychiatric disorders.

## Author Contributions

All authors contributed to writing the manuscript. All authors read and approved the submitted version.

## Conflict of Interest Statement

The authors declare that the research was conducted in the absence of any commercial or financial relationships that could be construed as a potential conflict of interest.

## References

[B1] Abbott R. Whear R. Nikolaou V. Bethel A. Coon J. T. Stein K. (2015). Tumour necrosis factor-alpha inhibitor therapy in chronic physical illness: a systematic review and meta-analysis of the effect on depression and anxiety. *J. Psychosom. Res.* 79 175–184. 10.1016/j.jpsychores.2015.04.008 25935351

[B2] Abekhoukh S. Sahin H. B. Grossi M. Zongaro S. Maurin T. Madrigal I. (2017). New insights into the regulatory function of CYFIP1 in the context of WAVE- and FMRP-containing complexes. *Dis. Model Mech.* 10 463–474. 10.1242/dmm.025809 28183735 PMC5399562

[B3] Abelaira H. M. Reus G. Z. Ignacio Z. M. Dos Santos M. A. de Moura A. B. Matos D. (2017). Effects of ketamine administration on mTOR and reticulum stress signaling pathways in the brain after the infusion of rapamycin into prefrontal cortex. *J. Psychiatr. Res.* 87 81–87. 10.1016/j.jpsychires.2016.12.002 28017918

[B4] Abrahams B. S. Geschwind D. H. (2008). Advances in autism genetics: on the threshold of a new neurobiology. *Nat. Rev. Genet.* 9 341–355. 10.1038/nrg2346 18414403 PMC2756414

[B5] Aguilar-Valles A. Haji N. De Gregorio D. Matta-Camacho E. Eslamizade M. J. Popic J. (2018). Translational control of depression-like behavior via phosphorylation of eukaryotic translation initiation factor 4E. *Nat. Commun.* 9:2459. 10.1038/s41467-018-04883-5 29941989 PMC6018502

[B6] Aguilar-Valles A. Matta-Camacho E. Khoutorsky A. Gkogkas C. Nader K. Lacaille J. C. (2015). Inhibition of group I metabotropic glutamate receptors reverses autistic-like phenotypes caused by deficiency of the translation repressor eIF4E binding protein 2. *J. Neurosci.* 35 11125–11132. 10.1523/JNEUROSCI.4615-14.2015 26245973 PMC4524980

[B7] Amorim I. S. Kedia S. Kouloulia S. Simbriger K. Gantois I. Jafarnejad S. M. (2018). Loss of eIF4E phosphorylation engenders depression-like behaviors via selective mRNA translation. *J. Neurosci.* 38 2118–2133. 10.1523/JNEUROSCI.2673-17.2018 29367404 PMC5824745

[B8] Baio J. Wiggins L. Christensen D. L. Maenner M. J. Daniels J. Warren Z. (2018). Prevalence of autism spectrum disorder among children aged 8 years – Autism and developmental disabilities monitoring network, 11 sites, United States, 2014. *MMWR Surveill. Summ.* 67 1–23. 10.15585/mmwr.ss6706a1 29701730 PMC5919599

[B9] Bear M. F. (2005). Therapeutic implications of the mGluR theory of fragile X mental retardation. *Genes Brain Behav.* 4 393–398. 10.1111/j.1601-183X.2005.00135.x 16098137

[B10] Bilousova T. V. Dansie L. Ngo M. Aye J. Charles J. R. Ethell D. W. (2009). Minocycline promotes dendritic spine maturation and improves behavioural performance in the fragile X mouse model. *J. Med. Genet.* 46 94–102. 10.1136/jmg.2008.061796 18835858

[B11] Bozdagi O. Sakurai T. Dorr N. Pilorge M. Takahashi N. Buxbaum J. D. (2012). Haploinsufficiency of Cyfip1 produces fragile X-like phenotypes in mice. *PLoS One* 7:e42422. 10.1371/journal.pone.0042422 22900020 PMC3416859

[B12] Bradshaw N. J. Porteous D. J. (2012). DISC1-binding proteins in neural development, signalling and schizophrenia. *Neuropharmacology* 62 1230–1241. 10.1016/j.neuropharm.2010.12.027 21195721 PMC3275753

[B13] Bramham C. R. Jensen K. B. Proud C. G. (2016). Tuning specific translation in cancer metastasis and synaptic memory: control at the MNK-eIF4E Axis. *Trends Biochem. Sci.* 41 847–858. 10.1016/j.tibs.2016.07.008 27527252

[B14] Brennand K. Savas J. N. Kim Y. Tran N. Simone A. Hashimoto-Torii K. (2015). Phenotypic differences in hiPSC NPCs derived from patients with schizophrenia. *Mol. Psychiatry* 20 361–368. 10.1038/mp.2014.22 24686136 PMC4182344

[B15] Cao R. Gkogkas C. G. de Zavalia N. Blum I. D. Yanagiya A. Tsukumo Y. (2015). Light-regulated translational control of circadian behavior by eIF4E phosphorylation. *Nat. Neurosci.* 18 855–862. 10.1038/nn.4010 25915475 PMC4446158

[B16] Carter M. T. Scherer S. W. (2013). Autism spectrum disorder in the genetics clinic: a review. *Clin. Genet.* 83 399–407. 10.1111/cge.12101 23425232

[B17] Chandran A. Iyo A. H. Jernigan C. S. Legutko B. Austin M. C. Karolewicz B. (2013). Reduced phosphorylation of the mTOR signaling pathway components in the amygdala of rats exposed to chronic stress. *Progr. Neuro-Psychopharmacol. Biol. Psychiatry* 40 240–245. 10.1016/j.pnpbp.2012.08.001 22889863 PMC3519947

[B18] Chen Z. Borek D. Padrick S. B. Gomez T. S. Metlagel Z. Ismail A. M. (2010). Structure and control of the actin regulatory WAVE complex. *Nature* 468 533–538. 10.1038/nature09623 21107423 PMC3085272

[B19] Contractor A. Klyachko V. A. Portera-Cailliau C. (2015). Altered neuronal and circuit excitability in fragile X syndrome. *Neuron* 87 699–715. 10.1016/j.neuron.2015.06.017 26291156 PMC4545495

[B20] Costa-Mattioli M. Monteggia L. M. (2013). mTOR complexes in neurodevelopmental and neuropsychiatric disorders. *Nat. Neurosci.* 16 1537–1543. 10.1038/nn.3546 24165680

[B21] Dahlhaus R. (2018). Of men and mice: modeling the fragile X syndrome. *Front. Mol. Neurosci.* 11:41. 10.3389/fnmol.2018.00041 29599705 PMC5862809

[B22] Dahlhaus R. El-Husseini A. (2010). Altered neuroligin expression is involved in social deficits in a mouse model of the fragile X syndrome. *Behav. Brain Res.* 208 96–105. 10.1016/j.bbr.2009.11.019 19932134

[B23] Dahlhaus R. Hines R. M. Eadie B. D. Kannangara T. S. Hines D. J. Brown C. E. (2010). Overexpression of the cell adhesion protein neuroligin-1 induces learning deficits and impairs synaptic plasticity by altering the ratio of excitation to inhibition in the hippocampus. *Hippocampus* 20 305–322. 10.1002/hipo.20630 19437420

[B24] De Wolf V. Brison N. Devriendt K. Peeters H. (2013). Genetic counseling for susceptibility loci and neurodevelopmental disorders: the del15q11.2 as an example. *Am. J. Med. Genet. Part A* 161A, 2846–2854. 10.1002/ajmg.a.36209 24123946

[B25] Dowlati Y. Herrmann N. Swardfager W. Liu H. Sham L. Reim E. K. (2010). A meta-analysis of cytokines in major depression. *Biol. Psychiatry* 67 446–457. 10.1016/j.biopsych.2009.09.033 20015486

[B26] Dwivedi Y. Rizavi H. S. Roberts R. C. Conley R. C. Tamminga C. A. Pandey G. N. (2001). Reduced activation and expression of ERK1/2 MAP kinase in the post-mortem brain of depressed suicide subjects. *J. Neurochem.* 77 916–928. 10.1046/j.1471-4159.2001.00300.x 11331420

[B27] Dwivedi Y. Zhang H. (2016). Altered ERK1/2 signaling in the brain of learned helpless rats: relevance in vulnerability to developing stress-induced depression. *Neural Plast.* 2016:7383724. 10.1155/2016/7383724 26839717 PMC4709739

[B28] Dziembowska M. Pretto D. I. Janusz A. Kaczmarek L. Leigh M. J. Gabriel N. (2013). High MMP-9 activity levels in fragile X syndrome are lowered by minocycline. *Am. J. Med. Genet. Part A* 161 1897–1903. 10.1002/ajmg.a.36023 23824974

[B29] Elsabbagh M. Divan G. Koh Y. J. Kim Y. S. Kauchali S. Marcin C. (2012). Global prevalence of autism and other pervasive developmental disorders. *Autism Res.* 5 160–179. 10.1002/aur.239 22495912 PMC3763210

[B30] English J. A. Fan Y. Focking M. Lopez L. M. Hryniewiecka M. Wynne K. (2015). Reduced protein synthesis in schizophrenia patient-derived olfactory cells. *Transl. Psychiatry* 5:e663. 10.1038/tp.2015.119 26485547 PMC4930119

[B31] Euesden J. Matcham F. Hotopf M. Steer S. Cope A. P. Lewis C. M. (2017). The relationship between mental health, disease severity, and genetic risk for depression in early rheumatoid arthritis. *Psychos. Med.* 79 638–645. 10.1097/PSY.0000000000000462 28282363 PMC5638421

[B32] Eulalio A. Behm-Ansmant I. Izaurralde E. (2007). P bodies: at the crossroads of post-transcriptional pathways. *Nat. Rev. Mol. Cell Biol.* 8 9–22. 10.1038/nrm2080 17183357

[B33] Feoktistova K. Tuvshintogs E. Do A. Fraser C. S. (2013). Human eIF4E promotes mRNA restructuring by stimulating eIF4A helicase activity. *Proc. Natl. Acad. Sci. U.S.A.* 110 13339–13344. 10.1073/pnas.1303781110 23901100 PMC3746923

[B34] Furic L. Rong L. Larsson O. Koumakpayi I. H. Yoshida K. Brueschke A. (2010). eIF4E phosphorylation promotes tumorigenesis and is associated with prostate cancer progression. *Proc. Natl. Acad. Sci. U.S.A.* 107 14134–14139. 10.1073/pnas.1005320107 20679199 PMC2922605

[B35] Furuichi Y. LaFiandra A. Shatkin A. J. (1977). 5′-Terminal structure and mRNA stability. *Nature* 266 235–239. 10.1038/266235a0 557727

[B36] Furuichi Y. Miura K. (1975). A blocked structure at the 5′ terminus of mRNA from cytoplasmic polyhedrosis virus. *Nature* 253 374–375. 10.1038/253374a0 163011

[B37] Gabis L. V. Baruch Y. K. Jokel A. Raz R. (2011). Psychiatric and autistic comorbidity in fragile X syndrome across ages. *J. Child Neurol.* 26 940–948. 10.1177/0883073810395937 21527394

[B38] Gantois I. Khoutorsky A. Popic J. Aguilar-Valles A. Freemantle E. Cao R. (2017). Metformin ameliorates core deficits in a mouse model of fragile X syndrome. *Nat. Med.* 23 674–677. 10.1038/nm.4335 28504725

[B39] Genheden M. Kenney J. W. Johnston H. E. Manousopoulou A. Garbis S. D. Proud C. G. (2015). BDNF stimulation of protein synthesis in cortical neurons requires the MAP kinase-interacting kinase MNK1. *J. Neurosci.* 35 972–984. 10.1523/JNEUROSCI.2641-14.2015 25609615 PMC4300335

[B40] Gingras A. C. Raught B. Sonenberg N. (1999). eIF4 initiation factors: effectors of mRNA recruitment to ribosomes and regulators of translation. *Annu. Rev. Biochem.* 68 913–963. 10.1146/annurev.biochem.68.1.913 10872469

[B41] Gingras A. C. Raught B. Sonenberg N. (2001). Regulation of translation initiation by FRAP/mTOR. *Genes Dev.* 15 807–826. 10.1101/gad.887201 11297505

[B42] Gkogkas C. G. Khoutorsky A. Cao R. Jafarnejad S. M. Prager-Khoutorsky M. Giannakas N. (2014). Pharmacogenetic inhibition of eIF4E-dependent Mmp9 mRNA translation reverses fragile X syndrome-like phenotypes. *Cell Rep.* 9 1742–1755. 10.1016/j.celrep.2014.10.064 25466251 PMC4294557

[B43] Gkogkas C. G. Khoutorsky A. Ran I. Rampakakis E. Nevarko T. Weatherill D. B. (2013). Autism-related deficits via dysregulated eIF4E-dependent translational control. *Nature* 493 371–377. 10.1038/nature11628 23172145 PMC4133997

[B44] Gkogkas C. G. Sonenberg N. (2013). Translational control and autism-like behaviors. *Cell Logist.* 3:e24551. 10.4161/cl.24551 24516777 PMC3906422

[B45] Grzmil M. Morin P. Jr. Lino M. M. Merlo A. Frank S. (2011). MAP kinase-interacting kinase 1 regulates SMAD2-dependent TGF-beta signaling pathway in human glioblastoma. *Cancer Res.* 71 2392–2402. 10.1158/0008-5472.CAN-10-3112 21406405

[B46] Gururajan A. van den Buuse M. (2014). Is the mTOR-signalling cascade disrupted in Schizophrenia? *J. Neurochem.* 129 377–387. 10.1111/jnc.12622 24266366

[B47] Haghighat A. Mader S. Pause A. Sonenberg N. (1995). Repression of cap-dependent translation by 4E-binding protein 1: competition with p220 for binding to eukaryotic initiation factor-4E. *EMBO J.* 14 5701–5709. 10.1002/j.1460-2075.1995.tb00257.x 8521827 PMC394685

[B48] Hines R. M. Wu L. Hines D. J. Steenland H. Mansour S. Dahlhaus R. (2008). Synaptic imbalance, stereotypies, and impaired social interactions in mice with altered neuroligin 2 expression. *J. Neurosci.* 28 6055–6067. 10.1523/JNEUROSCI.0032-08.2008 18550748 PMC6670530

[B49] Hinnebusch A. G. Ivanov I. P. Sonenberg N. (2016). Translational control by 5′-untranslated regions of eukaryotic mRNAs. *Science (New York, NY)* 352 1413–1416. 10.1126/science.aad9868 27313038 PMC7422601

[B50] Hoang H. D. Graber T. E. Alain T. (2018). Battling for ribosomes: translational control at the forefront of the antiviral response. *J. Mol. Biol.* 430 1965–1992. 10.1016/j.jmb.2018.04.040 29746850

[B51] Hoeffer C. A. Sanchez E. Hagerman R. J. Mu Y. Nguyen D. V. Wong H. (2012). Altered mTOR signaling and enhanced CYFIP2 expression levels in subjects with fragile X syndrome. *Genes Brain Behav.* 11 332–341. 10.1111/j.1601-183X.2012.00768.x 22268788 PMC3319643

[B52] Huntley G. W. (2012). Synaptic circuit remodelling by matrix metalloproteinases in health and disease. *Nat. Rev. Neurosci.* 13 743–757. 10.1038/nrn3320 23047773 PMC4900464

[B53] Huynh T. N. Shah M. Koo S. Y. Faraud K. S. Santini E. Klann E. (2015). eIF4E/Fmr1 double mutant mice display cognitive impairment in addition to ASD-like behaviors. *Neurobiol. Dis.* 83 67–74. 10.1016/j.nbd.2015.08.016 26306459 PMC4674395

[B54] Ignacio Z. M. Reus G. Z. Arent C. O. Abelaira H. M. Pitcher M. R. Quevedo J. (2016). New perspectives on the involvement of mTOR in depression as well as in the action of antidepressant drugs. *Br. J. Clin. Pharmacol.* 82 1280–1290. 10.1111/bcp.12845 26613210 PMC5061805

[B55] Ingolia N. T. Ghaemmaghami S. Newman J. R. Weissman J. S. (2009). Genome-wide analysis in vivo of translation with nucleotide resolution using ribosome profiling. *Science (New York, NY)* 324 218–223. 10.1126/science.1168978 19213877 PMC2746483

[B56] Ingolia N. T. Hussmann J. A. Weissman J. S. (2018). Ribosome Profiling: global Views of Translation. *Cold Spring Harb. Perspect. Biol.* 10.1101/cshperspect.a032698 [Epub ahead of print]. 30037969 PMC6496350

[B57] Jamain S. Quach H. Betancur C. Rastam M. Colineaux C. Gillberg I. C. (2003). Mutations of the X-linked genes encoding neuroligins NLGN3 and NLGN4 are associated with autism. *Nat. Genet.* 34 27–29. 10.1038/ng1136 12669065 PMC1925054

[B58] Jan E. Mohr I. Walsh D. (2016). A cap-to-tail guide to mRNA translation strategies in virus-infected cells. *Annu. Rev. Virol.* 3 283–307. 10.1146/annurev-virology-100114-055014 27501262

[B59] Jernigan C. S. Goswami D. B. Austin M. C. Iyo A. H. Chandran A. Stockmeier C. A. (2011). The mTOR signaling pathway in the prefrontal cortex is compromised in major depressive disorder. *Progr. Neuro-Psychopharmacol. Biol. Psychiatry* 35 1774–1779. 10.1016/j.pnpbp.2011.05.010 21635931 PMC3154612

[B60] Joshi B. Cai A. L. Keiper B. D. Minich W. B. Mendez R. Beach C. M. (1995). Phosphorylation of eukaryotic protein synthesis initiation factor 4E at Ser-209. *J. Biol. Chem.* 270 14597–14603. 10.1074/jbc.270.24.14597 7782323

[B61] Joshi S. Platanias L. C. (2014). Mnk kinase pathway: cellular functions and biological outcomes. *World J. Biol. Chem.* 5 321–333. 10.4331/wjbc.v5.i3.321 25225600 PMC4160526

[B62] Jung H. Gkogkas C. G. Sonenberg N. Holt C. E. (2014). Remote control of gene function by local translation. *Cell* 157 26–40. 10.1016/j.cell.2014.03.005 24679524 PMC3988848

[B63] Kahn R. S. Sommer I. E. Murray R. M. Meyer-Lindenberg A. Weinberger D. R. Cannon T. D. (2015). Schizophrenia. *Nat. Rev. Dis. Primers* 1:15067. 10.1038/nrdp.2015.67 27189524

[B64] Kavanagh D. H. Dwyer S. O’Donovan M. C. Owen M. J. (2013). The ENCODE project: implications for psychiatric genetics. *Mol. Psychiatry* 18 540–542. 10.1038/mp.2013.13 23478746

[B65] Kearse M. G. Green K. M. Krans A. Rodriguez C. M. Linsalata A. E. Goldstrohm A. C. (2016). CGG repeat-associated non-AUG translation utilizes a cap-dependent scanning mechanism of initiation to produce toxic proteins. *Mol. Cell* 62 314–322. 10.1016/j.molcel.2016.02.034 27041225 PMC4854189

[B66] Kelleher R. J. III Bear M. F. (2008). The autistic neuron: troubled translation? *Cell* 135 401–406. 10.1016/j.cell.2008.10.017 18984149

[B67] Kelsoe J. R. (2004). Genomics and the human genome project: implications for psychiatry. *Int. Rev. Psychiatry (Abingdon, England)* 16 294–300. 10.1080/09540260400014385 16194762

[B68] Kim J. Y. Duan X. Liu C. Y. Jang M. H. Guo J. U. Pow-anpongkul N. (2009). DISC1 regulates new neuron development in the adult brain via modulation of AKT-mTOR signaling through KIAA1212. *Neuron* 63 761–773. 10.1016/j.neuron.2009.08.008 19778506 PMC3075620

[B69] Kirov G. Grozeva D. Norton N. Ivanov D. Mantripragada K. K. Holmans P. (2009). Support for the involvement of large copy number variants in the pathogenesis of schizophrenia. *Hum. Mol. Genet.* 18 1497–1503. 10.1093/hmg/ddp043 19181681 PMC2664144

[B70] Kirov G. Pocklington A. J. Holmans P. Ivanov D. Ikeda M. Ruderfer D. (2012). De novo CNV analysis implicates specific abnormalities of postsynaptic signalling complexes in the pathogenesis of schizophrenia. *Mol. Psychiatry* 17 142–153. 10.1038/mp.2011.154 22083728 PMC3603134

[B71] Knauf U. Tschopp C. Gram H. (2001). Negative regulation of protein translation by mitogen-activated protein kinase-interacting kinases 1 and 2. *Mol. Cell. Biol.* 21 5500–5511. 10.1128/MCB.21.16.5500-5511.2001 11463832 PMC87272

[B72] Konicek B. W. Stephens J. R. McNulty A. M. Robichaud N. Peery R. B. Dumstorf C. A. (2011). Therapeutic inhibition of MAP kinase interacting kinase blocks eukaryotic initiation factor 4E phosphorylation and suppresses outgrowth of experimental lung metastases. *Cancer Res.* 71 1849–1857. 10.1158/0008-5472.CAN-10-3298 21233335

[B73] Lachance P. E. Miron M. Raught B. Sonenberg N. Lasko P. (2002). Phosphorylation of eukaryotic translation initiation factor 4E is critical for growth. *Mol. Cell. Biol.* 22 1656–1663. 10.1128/MCB.22.6.1656-1663.2002 11865045 PMC135594

[B74] Li Z. Zhang Y. Ku L. Wilkinson K. D. Warren S. T. Feng Y. (2001). The fragile X mental retardation protein inhibits translation via interacting with mRNA. *Nucleic Acids Res.* 29 2276–2283. 10.1093/nar/29.11.2276 11376146 PMC55699

[B75] Liu X. L. Luo L. Mu R. H. Liu B. B. Geng D. Liu Q. (2015). Fluoxetine regulates mTOR signalling in a region-dependent manner in depression-like mice. *Sci. Rep.* 5:16024. 10.1038/srep16024 26522512 PMC4629199

[B76] Lombardo M. V. Moon H. M. Su J. Palmer T. D. Courchesne E. Pramparo T. (2018). Maternal immune activation dysregulation of the fetal brain transcriptome and relevance to the pathophysiology of autism spectrum disorder. *Mol. Psychiatry* 23 1001–1013. 10.1038/mp.2017.15 28322282 PMC5608645

[B77] Mackay-Sim A. (2012). Concise review: patient-derived olfactory stem cells: new models for brain diseases. *Stem Cells (Dayton, Ohio)* 30 2361–2365. 10.1002/stem.1220 22961669

[B78] Magnuson K. M. Constantino J. N. (2011). Characterization of depression in children with autism spectrum disorders. *J. Dev. Behav. Pediatr. JDBP* 32 332–340. 10.1097/DBP.0b013e318213f56c 21502871 PMC3154372

[B79] Malhotra D. McCarthy S. Michaelson J. J. Vacic V. Burdick K. E. Yoon S. (2011). High frequencies of de novo CNVs in bipolar disorder and schizophrenia. *Neuron* 72 951–963. 10.1016/j.neuron.2011.11.007 22196331 PMC3921625

[B80] Malina A. Cencic R. Pelletier J. (2011). Targeting translation dependence in cancer. *Oncotarget* 2 76–88. 10.18632/oncotarget.218 21378410 PMC3248143

[B81] Malina A. Mills J. R. Pelletier J. (2012). Emerging therapeutics targeting mRNA translation. *Cold Spring Harb. Perspect. Biol.* 4:a012377. 10.1101/cshperspect.a012377 22474009 PMC3312682

[B82] Malka-Mahieu H. Newman M. Desaubry L. Robert C. Vagner S. (2017). Molecular pathways: the eIF4F translation initiation complex-new opportunities for cancer treatment. *Clin. Cancer Res.* 23 21–25. 10.1158/1078-0432.CCR-14-2362 27789529

[B83] McCary L. M. Roberts J. E. (2013). Early identification of autism in fragile X syndrome: a review. *J. Intell. Disabil. Res.* 57 803–814. 10.1111/j.1365-2788.2012.01609.x 22974167 PMC4023162

[B84] McKendrick L. Morley S. J. Pain V. M. Jagus R. Joshi B. (2001). Phosphorylation of eukaryotic initiation factor 4E (eIF4E) at Ser209 is not required for protein synthesis in vitro and in vivo. *Eur. J. Biochem.* 268 5375–5385. 10.1046/j.0014-2956.2001.02478.x 11606200

[B85] Michopoulos V. Rothbaum A. O. Jovanovic T. Almli L. M. Bradley B. Rothbaum B. O. (2015). Association of CRP genetic variation and CRP level with elevated PTSD symptoms and physiological responses in a civilian population with high levels of trauma. *Am. J. Psychiatry* 172 353–362. 10.1176/appi.ajp.2014.14020263 25827033 PMC4440454

[B86] Miller A. H. Raison C. L. (2016). The role of inflammation in depression: from evolutionary imperative to modern treatment target. *Nat. Rev. Immunol.* 16 22–34. 10.1038/nri.2015.5 26711676 PMC5542678

[B87] Moerke N. J. Aktas H. Chen H. Cantel S. Reibarkh M. Y. Fahmy A. (2007). Small-molecule inhibition of the interaction between the translation initiation factors eIF4E and eIF4G. *Cell* 128 257–267. 10.1016/j.cell.2006.11.046 17254965

[B88] Mostafavi S. Battle A. Zhu X. Potash J. B. Weissman M. M. Shi J. (2014). Type I interferon signaling genes in recurrent major depression: increased expression detected by whole-blood RNA sequencing. *Mol. Psychiatry* 19 1267–1274. 10.1038/mp.2013.161 24296977 PMC5404932

[B89] Mukhopadhyay R. Jia J. Arif A. Ray P. S. Fox P. L. (2009). The GAIT system: a gatekeeper of inflammatory gene expression. *Trends Biochem. Sci.* 34 324–331. 10.1016/j.tibs.2009.03.004 19535251 PMC3637685

[B90] Müller N. (2018). “Chapter 28 - Clinical trials of anti-inflammatory treatments of major depression,” in *Inflammation and Immunity in Depression*, ed. Baune B. T. (Cambridge: Academic Press), 489–507.

[B91] Napoli I. Mercaldo V. Boyl P. P. Eleuteri B. Zalfa F. De Rubeis S. (2008). The fragile X syndrome protein represses activity-dependent translation through CYFIP1, a new 4E-BP. *Cell* 134 1042–1054. 10.1016/j.cell.2008.07.031 18805096

[B92] Neves-Pereira M. Muller B. Massie D. Williams J. H. O’Brien P. C. Hughes A. (2009). Deregulation of EIF4E: a novel mechanism for autism. *J. Med. Genet.* 46 759–765. 10.1136/jmg.2009.066852 19556253

[B93] Nievergelt C. M. Ashley-Koch A. E. Dalvie S. Hauser M. A. Morey R. A. Smith A. K. (2018). Genomic approaches to posttraumatic stress disorder: the psychiatric genomic consortium initiative. *Biol. Psychiatry* 83 831–839. 10.1016/j.biopsych.2018.01.020 29555185 PMC5915904

[B94] Oguro-Ando A. Rosensweig C. Herman E. Nishimura Y. Werling D. Bill B. R. (2015). Increased CYFIP1 dosage alters cellular and dendritic morphology and dysregulates mTOR. *Mol. Psychiatry* 20 1069–1078. 10.1038/mp.2014.124 25311365 PMC4409498

[B95] Panja D. Kenney J. W. D’Andrea L. Zalfa F. Vedeler A. Wibrand K. (2014). Two-stage translational control of dentate gyrus LTP consolidation is mediated by sustained BDNF-TrkB signaling to MNK. *Cell Rep.* 9 1430–1445. 10.1016/j.celrep.2014.10.016 25453757

[B96] Park S. W. Lee J. G. Seo M. K. Lee C. H. Cho H. Y. Lee B. J. (2014). Differential effects of antidepressant drugs on mTOR signalling in rat hippocampal neurons. *Int. J. Neuropsychopharmacol.* 17 1831–1846. 10.1017/S1461145714000534 24901414

[B97] Pathania M. Davenport E. C. Muir J. Sheehan D. F. Lopez-Domenech G. Kittler J. T. (2014). The autism and schizophrenia associated gene CYFIP1 is critical for the maintenance of dendritic complexity and the stabilization of mature spines. *Transl. Psychiatry* 4:e374. 10.1038/tp.2014.16 24667445 PMC3966042

[B98] Pause A. Belsham G. J. Gingras A. C. Donze O. Lin T. A. Lawrence J. C. (1994). Insulin-dependent stimulation of protein synthesis by phosphorylation of a regulator of 5′-cap function. *Nature* 371 762–767. 10.1038/371762a0 7935836

[B99] Pelletier J. Sonenberg N. (1987). The involvement of mRNA secondary structure in protein synthesis. *Biochem. Cell Biol.* 65 576–581. 10.1139/o87-074 3322328

[B100] Pettem K. L. Yokomaku D. Takahashi H. Ge Y. Craig A. M. (2013). Interaction between autism-linked MDGAs and neuroligins suppresses inhibitory synapse development. *J. Cell Biol.* 200 321–336. 10.1083/jcb.201206028 23358245 PMC3563690

[B101] Pyronnet S. Imataka H. Gingras A. C. Fukunaga R. Hunter T. Sonenberg N. (1999). Human eukaryotic translation initiation factor 4G (eIF4G) recruits mnk1 to phosphorylate eIF4E. *EMBO J.* 18 270–279. 10.1093/emboj/18.1.270 9878069 PMC1171121

[B102] Rao J. S. Ertley R. N. Lee H. J. DeMar J. C. Jr. Arnold J. T. (2007). n-3 polyunsaturated fatty acid deprivation in rats decreases frontal cortex BDNF via a p38 MAPK-dependent mechanism. *Mol. Psychiatry* 12 36–46. 10.1038/sj.mp.4001888 16983391

[B103] Rhoads R. E. Joshi-Barve S. Rinker-Schaeffer C. (1993). Mechanism of action and regulation of protein synthesis initiation factor 4E: effects on mRNA discrimination, cellular growth rate, and oncogenesis. *Prog Nucleic Acid Res. Mol. Biol.* 46 183–219. 10.1016/S0079-6603(08)61022-3 8234784

[B104] Richter J. D. Sonenberg N. (2005). Regulation of cap-dependent translation by eIF4E inhibitory proteins. *Nature* 433 477–480. 10.1038/nature03205 15690031

[B105] Roberts R. C. (2007). Schizophrenia in translation: disrupted in schizophrenia (DISC1): integrating clinical and basic findings. *Schizophr. Bull.* 33 11–15. 10.1093/schbul/sbl063 17138582 PMC2632285

[B106] Robichaud N. del Rincon S. V. Huor B. Alain T. Petruccelli L. A. Hearnden J. (2015). Phosphorylation of eIF4E promotes EMT and metastasis via translational control of SNAIL and MMP-3. *Oncogene* 34 2032–2042. 10.1038/onc.2014.146 24909168 PMC4978545

[B107] Rothwell P. E. Fuccillo M. V. Maxeiner S. Hayton S. J. Gokce O. Lim B. K. (2014). Autism-associated neuroligin-3 mutations commonly impair striatal circuits to boost repetitive behaviors. *Cell* 158 198–212. 10.1016/j.cell.2014.04.045 24995986 PMC4120877

[B108] Rotschafer S. E. Trujillo M. S. Dansie L. E. Ethell I. M. Razak K. A. (2012). Minocycline treatment reverses ultrasonic vocalization production deficit in a mouse model of Fragile X Syndrome. *Brain Res.* 1439 7–14. 10.1016/j.brainres.2011.12.041 22265702

[B109] Sandin S. Lichtenstein P. Kuja-Halkola R. Hultman C. Larsson H. Reichenberg A. (2017). The heritability of autism spectrum disorder. *JAMA* 318:1182. 10.1001/jama.2017.12141 28973605 PMC5818813

[B110] Santini E. Huynh T. N. Longo F. Koo S. Y. Mojica E. D’Andrea L. (2017). Reducing eIF4E-eIF4G interactions restores the balance between protein synthesis and actin dynamics in fragile X syndrome model mice. *Sci. Signal.* 10:eaan0665. 29114037 10.1126/scisignal.aan0665 PMC5858943

[B111] Santini E. Huynh T. N. MacAskill A. F. Carter A. G. Pierre P. Ruggero D. (2013). Exaggerated translation causes synaptic and behavioural aberrations associated with autism. *Nature* 493 411–415. 10.1038/nature11782 23263185 PMC3548017

[B112] Santini E. Klann E. (2014). Reciprocal signaling between translational control pathways and synaptic proteins in autism spectrum disorders. *Sci. Signal.* 7 1–11. 10.1126/scisignal.2005832 25351249 PMC6002803

[B113] Santoro M. R. Bray S. M. Warren S. T. (2012). Molecular mechanisms of fragile X syndrome: a twenty-year perspective. *Annu. Rev. Pathol.* 7 219–245. 10.1146/annurev-pathol-011811-132457 22017584

[B114] Saxton R. A. Sabatini D. M. (2017). mTOR Signaling in growth, metabolism, and disease. *Cell* 169 361–371. 10.1016/j.cell.2017.03.035 28388417

[B115] Shatkin A. J. (1976). Capping of eucaryotic mRNAs. *Cell* 9 645–653. 10.1016/0092-8674(76)90128-8 1017010

[B116] Shelton R. C. Claiborne J. Sidoryk-Wegrzynowicz M. Reddy R. Aschner M. Lewis D. A. (2011). Altered expression of genes involved in inflammation and apoptosis in frontal cortex in major depression. *Mol. Psychiatry* 16 751–762. 10.1038/mp.2010.52 20479761 PMC2928407

[B117] Shine J. Dalgarno L. (1975). Determinant of cistron specificity in bacterial ribosomes. *Nature* 254 34–38. 10.1038/254034a0 803646

[B118] Shveygert M. Kaiser C. Bradrick S. S. Gromeier M. (2010). Regulation of eukaryotic initiation factor 4E (eIF4E) phosphorylation by mitogen-activated protein kinase occurs through modulation of Mnk1-eIF4G interaction. *Mol. Cell. Biol.* 30 5160–5167. 10.1128/MCB.00448-10 20823271 PMC2953056

[B119] Sidhu H. Dansie L. E. Hickmott P. W. Ethell D. W. Ethell I. M. (2014). Genetic removal of matrix metalloproteinase 9 rescues the symptoms of fragile X syndrome in a mouse model. *J. Neurosci.* 34 9867–9879. 10.1523/JNEUROSCI.1162-14.2014 25057190 PMC4107404

[B120] Sonenberg N. Hinnebusch A. G. (2007). New modes of translational control in development, behavior, and disease. *Mol. Cell* 28 721–729. 10.1016/j.molcel.2007.11.018 18082597

[B121] Sonenberg N. Hinnebusch A. G. (2009). Regulation of translation initiation in eukaryotes: mechanisms and biological targets. *Cell* 136 731–745. 10.1016/j.cell.2009.01.042 19239892 PMC3610329

[B122] Sonenberg N. Rupprecht K. M. Hecht S. M. Shatkin A. J. (1979). Eukaryotic mRNA cap binding protein: purification by affinity chromatography on sepharose-coupled m7GDP. *Proc. Natl. Acad. Sci. U.S.A.* 76 4345–4349. 10.1073/pnas.76.9.4345 291969 PMC411571

[B123] St Clair D. Johnstone M. (2018). Using mouse transgenic and human stem cell technologies to model genetic mutations associated with schizophrenia and autism. *Philos. Trans. R. Soc. Lond. B Biol. Sci.* 373 20170037. 10.1098/rstb.2017.0037 29352035 PMC5790834

[B124] Stefansson H. Rujescu D. Cichon S. Pietilainen O. P. Ingason A. Steinberg S. (2008). Large recurrent microdeletions associated with schizophrenia. *Nature* 455 232–236. 10.1038/nature07229 18668039 PMC2687075

[B125] Steinberger J. Chu J. Maiga R. I. Sleiman K. Pelletier J. (2017). Developing anti-neoplastic biotherapeutics against eIF4F. *Cell Mol. Life. Sci.* 74 1681–1692. 10.1007/s00018-016-2430-8 28004147 PMC11107644

[B126] Sullivan P. F. Daly M. J. O’Donovan M. (2012). Genetic architectures of psychiatric disorders: the emerging picture and its implications. *Nat. Rev. Genet.* 13 537–551. 10.1038/nrg3240 22777127 PMC4110909

[B127] Todd P. K. Oh S. Y. Krans A. He F. Sellier C. Frazer M. (2013). CGG repeat-associated translation mediates neurodegeneration in fragile X tremor ataxia syndrome. *Neuron* 78 440–455. 10.1016/j.neuron.2013.03.026 23602499 PMC3831531

[B128] Topol A. English J. A. Flaherty E. Rajarajan P. Hartley B. J. Gupta S. (2015). Increased abundance of translation machinery in stem cell-derived neural progenitor cells from four schizophrenia patients. *Transl. Psychiatry* 5:e662. 10.1038/tp.2015.118 26485546 PMC4930118

[B129] Truitt M. L. Conn C. S. Shi Z. Pang X. Tokuyasu T. Coady A. M. (2015). Differential requirements for eIF4E dose in normal development and cancer. *Cell* 162 59–71. 10.1016/j.cell.2015.05.049 26095252 PMC4491046

[B130] Ueda T. Watanabe-Fukunaga R. Fukuyama H. Nagata S. Fukunaga R. (2004). Mnk2 and Mnk1 are essential for constitutive and inducible phosphorylation of eukaryotic initiation factor 4E but not for cell growth or development. *Mol. Cell. Biol.* 24 6539–6549. 10.1128/MCB.24.15.6539-6549.2004 15254222 PMC444855

[B131] Walsh T. McClellan J. M. McCarthy S. E. Addington A. M. Pierce S. B. Cooper G. M. (2008). Rare structural variants disrupt multiple genes in neurodevelopmental pathways in schizophrenia. *Science (New York, NY)* 320 539–543. 10.1126/science.1155174 18369103

[B132] Waltes R. Gfesser J. Haslinger D. Schneider-Momm K. Biscaldi M. Voran A. (2014). Common EIF4E variants modulate risk for autism spectrum disorders in the high-functioning range. *J. Neural. Transm.* 121 1107–1116. 10.1007/s00702-014-1230-2 24818597

[B133] Wiedlocha M. Marcinowicz P. Krupa R. Janoska-Jazdzik M. Janus M. Debowska W. (2018). Effect of antidepressant treatment on peripheral inflammation markers – A meta-analysis. *Progr. Neuro-Psychopharmacol. Biol. Psychiatry* 80 217–226. 10.1016/j.pnpbp.2017.04.026 28445690

[B134] Xu B. Roos J. L. Levy S. van Rensburg E. J. Gogos J. A. Karayiorgou M. (2008). Strong association of de novo copy number mutations with sporadic schizophrenia. *Nat. Genet.* 40 880–885. 10.1038/ng.162 18511947

[B135] Yang G. Smibert C. A. Kaplan D. R. Miller F. D. (2014). An eIF4E1/4E-T complex determines the genesis of neurons from precursors by translationally repressing a proneurogenic transcription program. *Neuron* 84 723–739. 10.1016/j.neuron.2014.10.022 25456498

[B136] Yonan A. L. Alarcón M. Cheng R. Magnusson P. K. E. Spence S. J. Palmer A. A. (2003). A genomewide screen of 345 families for autism-susceptibility loci. *Am. J. Hum. Genet.* 73 886–897. 10.1086/378778 13680528 PMC1180610

[B137] Yoon K. J. Nguyen H. N. Ursini G. Zhang F. Kim N. S. Wen Z. (2014). Modeling a genetic risk for schizophrenia in iPSCs and mice reveals neural stem cell deficits associated with adherens junctions and polarity. *Cell Stem Cell* 15 79–91. 10.1016/j.stem.2014.05.003 24996170 PMC4237009

[B138] Zahr S. K. Yang G. Kazan H. Borrett M. J. Yuzwa S. A. Voronova A. (2018). A translational repression complex in developing mammalian neural stem cells that regulates neuronal specification. *Neuron* 97 520.e526–537.e526. 10.1016/j.neuron.2017.12.045 29395907

[B139] Zhou M. Li W. Huang S. Song J. Kim J. Y. Tian X. (2013). mTOR Inhibition ameliorates cognitive and affective deficits caused by Disc1 knockdown in adult-born dentate granule neurons. *Neuron* 77 647–654. 10.1016/j.neuron.2012.12.033 23439118 PMC3586374

[B140] Zhou Y. Cao Z. Yang M. Xi X. Guo Y. Fang M. (2017). Comorbid generalized anxiety disorder and its association with quality of life in patients with major depressive disorder. *Sci. Rep.* 7:40511. 10.1038/srep40511 28098176 PMC5241829

[B141] Zhuang F. Li M. Gao X. Wang Y. Wang D. Ma X. (2016). The antidepressant-like effect of alarin is related to TrkB-mTOR signaling and synaptic plasticity. *Behav. Brain Res.* 313 158–171. 10.1016/j.bbr.2016.06.057 27374162

